# The importance and challenges of shared decision making in older people with multimorbidity

**DOI:** 10.1371/journal.pmed.1002530

**Published:** 2018-03-13

**Authors:** Tammy Hoffmann, Jesse Jansen, Paul Glasziou

**Affiliations:** 1 Centre for Research in Evidence-Based Practice, Faculty of Health Sciences and Medicine, Bond University, Robina, Australia; 2 Wiser Healthcare Program, Sydney School of Public Health, The University of Sydney, Sydney, Australia; 3 Centre for Medical Psychology and Evidence-based Decision-making (CeMPED), The University of Sydney, Sydney, Australia

## Abstract

In a Perspective, Tammy Hoffmann and colleagues discuss medical decision making for elderly patients with multimorbidity.

Typically, shared decision making, with or without a decision aid, involves patients in discussions about the options for treatment, the benefits and harms of each therapy, and the patient’s preferences, and a collaborative decision about how to proceed is made. For single conditions, that requires some motivation and teachable skills. But an additional challenge in providing healthcare for older people is the likelihood of multimorbidity: approximately half of older adults have 3 or more chronic conditions [1]. The patterns of comorbidity are largely determined by common conditions in older people, such as coronary heart disease, diabetes, hypertension, chronic obstructive pulmonary disease (COPD), heart failure, depression, arthritis, and cancer. Multiple conditions complicate shared decision making, as management is not the simple sum of the parts. Should a patient with late-stage cancer continue statins? When is an implantable defibrillator appropriate in a patient with dementia? Are beta blockers mandatory in a patient who is depressed after a myocardial infarction? Multimorbidity is a problem itself but also creates interactions that can generate additional problems—of particular concern are polypharmacy and burden of treatment.

## Polypharmacy

Most clinical practice guidelines focus on a single disease [2]. Applying single-disease guidelines for multiple conditions increases the risk of polypharmacy, which is the use of multiple prescribed medications (sometimes defined as 5 or more) by a patient. In turn, this increases the risk of adverse drug reactions and interactions and complicates predicting the effect of each individual drug to compare overall benefits and harms [1,2]. The body of evidence informing guidelines is also problematic. For example, clinical trials often do not measure the outcomes with higher priority for many older adults, such as independent living and quality of life [1]. Additionally, as older people are often excluded from clinical trials, evidence of the benefits and harms of treatments and tests for this group is often limited, and trial populations may not adequately represent community populations [3]. Guideline recommendations rarely acknowledge such limitations or provide information about evidence uncertainty or characteristics of the patient group upon which the evidence is based, and this hinders extrapolation [4]. Combined with clinicians’ tendency to do something rather than nothing, this contributes to the problem of polypharmacy.

## Burden of treatment

A high ‘burden of treatment’—such as healthcare visits, refilling prescriptions, diet, and self-managing care—on multimorbid patients is common, especially if all recommendations of guidelines are followed. Boyd and colleagues [2] highlighted that if the care for a hypothetical woman aged 79 years with 5 conditions (hypertension, diabetes, osteoporosis, osteoarthritis, and chronic obstructive pulmonary disease) followed guidelines, she would be prescribed 12 medications (19 doses per day), all with their own specific instructions; have 14 nondrug tasks; and see a variety of clinicians. Such a complex treatment plan is unlikely to be feasible, and the hard work it requires can place a significant demand on people’s lives [2,5].

## Principles for handling multimorbidity in older people

The Ariadne principles are a set of guiding principles for how to handle multimorbidity, particularly in primary care consultations, with the aim of reducing the burden of treatment and disease [6]. At their core is the sharing of realistic treatment goals that have been collaboratively decided by physicians and patients. That involves 2 phases: assessing priorities and then deciding among the management options for the highest priority problems.

Of course, for many older people, particularly those who have cognitive impairment, the decision-making process will likely also involve family members or other informal carers. Consultation with and communication between providers is also essential when caring for older people with multimorbidity. There is a valuable role for generalist care, whether that be provided by a general practitioner, geriatrician, or physician. It is important to periodically monitor the priorities and decisions that have been made to ensure the management plan is still of benefit to the patient, is in line with preferences and circumstances, and remains feasible [1]. In older people with multimorbidity, circumstances and preferences can change rapidly because of the occurrence of events such as hospitalisations [6]. A generalist can provide the patient-centred continuity of care that situations like this require [1].

## Problem prioritisation, goal setting, and shared decision making

Key to providing patient-centred care is careful and shared decision making. Problem and decision prioritisation should involve identifying the patient’s primary concerns, priorities, and preferences and subsequently engaging in patient-centred goal setting [1,6]. In an examination of the health outcome priorities of older adults with hypertension and fall risk, half of the participants identified reducing the risk of cardiovascular events as more important than reducing their combined risk of fall injuries or medication symptoms, whereas the other half of the participants identified the opposite priorities [7].

Once goals have been collaboratively decided, the conversation shifts to the highest priority issue and determining how this goal may best be attained. Ideally, this involves discussing the treatment options—and, for each option, the benefits and harms (including the size or likelihood of each, individualised to the patient where possible) and the practicalities and feasibility (including the treatment burden and costs) of the options—and then, the patient’s preferences about the options [8]. Many of these tasks are part of a process known as shared decision making [8]. Part of the progression to making an informed choice in which the decision aligns with the patient’s preferences and values involves the patient weighing up the likely benefit–harm trade-off. For example, older people’s willingness to take medication for primary prevention of cardiovascular disease is more sensitive to the adverse effects than it is to the benefits [9]. There are likely to be different benefit–harm trade-offs for each individual older patient with multimorbidity because of the heterogeneity within this group. Patients vary widely in their health and function (both physical and cognitive), tolerance of side effects, life expectancy, and treatment and health outcome preferences, such as valuing length of life versus quality of life [1]. In the situation of older people with multimorbidity, among the additional knowledge and expertise that the clinician contributes to the decision-making process is the potential for adverse drug–disease interactions and drug–drug interactions.

## Barriers when using existing guidelines and decisions aids to achieve appropriate care

Guidelines can sometimes be an obstacle in managing multimorbidity. Most existing evidence and decision-support tools do not adequately facilitate shared decision making, let alone for older people and those with multimorbidity. For example, some guidelines on heart failure and primary cardiovascular disease prevention mostly focus on risk reduction, without considering patients’ concerns or preferences (such as about the quality of their remaining life) and without acknowledging the need for patient-centred goal setting [10,11]. Related to this, guidelines typically have a strong focus on starting medication, with limited guidance about not starting, reducing, or stopping medication (known as ‘deprescribing’) [10]. Guidelines often provide blanket statements around the importance of using ‘clinical judgement’ in these more complex circumstances and how ‘decisions should be made with the patient, reflecting his or her preferences, needs, and values’ [10,12]. Most guidelines, however, do not offer specific guidance, approaches, or tools on how to achieve this [4,12] and are not designed in a way that optimally supports patient involvement and shared decision making.

Patient decision aids are tools aimed at supporting the decision-making process. They typically contain synthesised information and evidence about the options, the benefits and harms, and sometimes questions that prompt the patient to think about preferences and values. While decision aids are available for some decisions about cardiovascular conditions, as with most guidelines, they are not designed for people with multimorbidity. Although a systematic review of studies evaluating the use of decision aids by older people showed that decision aids have the potential to improve patient knowledge, risk perception, and decision participation and decrease decisional conflict, similar to the effects of decision aids in a general population, few decision aids are developed specifically for the elderly [13]. Consequently, most aids are not tailored to the older patient context and do not address multimorbidity, family or carer involvement, or the cognitive and emotional changes that might impact on decision making; nor are they validated in older-old adults (aged >80 years) [13]. Hence, it is unclear how useful existing decision aids are for helping clinicians to achieve shared decision making with older people who have multimorbidity.

It is unlikely that existing approaches to developing and formatting decision aids (or a single decision aid) could adequately incorporate the relevant evidence for multiple chronic conditions, decisions, and the potential interactions and combinations. As guidelines have started to emerge that are not about single diseases but are focussed on optimising care for people with multimorbidity (e.g., the United Kingdom National Institute for Health and Care Excellence [NICE] Guideline NG56, ‘Multimorbidity: Clinical assessment and management’), a more flexible approach to decision-making and generic tools may also be valuable. For example, generic versions of decision-support tools are available, such as the Ottawa Personal Decision Guide [14] (a template that prompts the clinician and patient to discuss and complete a form with the options, their pros and cons, how much each pro or con matters to the patient, available support, further decision-making needs, and next steps), alongside clinical encounter tools that aim to increase patient involvement and support meaningful conversations about goals and priorities in the context of multimorbidity [15].

Shared decision making in older adults with multimorbidity needs a stepwise and individualised approach. The sharing first needs to occur in a discussion that considers what the patient’s priority problems and goals are so that any care provided aligns with these. For the decisions about how to achieve the prioritised goals, the more typical ‘sharing of the decision’ about the preferred option can then occur, although existing decision aids should be used with caution as they are unlikely to account for the increased risk of harm in older people or potential drug–drug or drug–condition interactions, and personalisation of this information is ideally needed. In juggling the competing priorities and conditions (and clinicians), our aim should be to help patients achieve their goals as much as possible while disrupting their lives as little as possible.

## Abbreviations

COPD: chronic obstructive pulmonary disease
NICE: National Institute for Health and Care Excellence
